# Change of Floral Orientation within an Inflorescence Affects Pollinator Behavior and Pollination Efficiency in a Bee-Pollinated Plant, *Corydalis sheareri*

**DOI:** 10.1371/journal.pone.0095381

**Published:** 2014-04-17

**Authors:** Hui Wang, Shuang Tie, Dan Yu, You-Hao Guo, Chun-Feng Yang

**Affiliations:** 1 College of Life Sciences, Wuhan University, Wuhan, China; 2 Key Laboratory of Aquatic Botany and Watershed Ecology, Wuhan Botanical Garden, Chinese Academy of Sciences, Wuhan, China; University of Arizona, United States of America

## Abstract

Vertical raceme or spike inflorescences that are bee-pollinated tend to present their flowers horizontally. Horizontal presentation of flowers is hypothesized to enhance pollinator recognition and pollination precision, and it may also ensure greater consistency of pollinator movement on inflorescences. We tested the hypotheses using bee-pollinated *Corydalis sheareri* which has erect inflorescences consisting of flowers with horizontal orientation. We altered the orientation of individual flowers and prepared three types of inflorescences: (i) unmanipulated inflorescences with horizontal-facing flowers, (ii) inflorescences with flowers turned upward, and (iii) inflorescences with flowers turned downward. We compared number of inflorescences approached and visited, number of successive probes within an inflorescence, the direction percentage of vertical movement on inflorescences, efficiency of pollen removal and seed production per inflorescence. Deviation from horizontal orientation decreased both approaches and visits by leafcutter bees and bumble bees to inflorescences. Changes in floral orientation increased the proportion of downward movements by leafcutter bees and decreased the consistency of pollinator movement on inflorescences. In addition, pollen removal per visit and seed production per inflorescence also declined with changes of floral orientation. In conclusion, floral orientation seems more or less optimal as regards bee behavior and pollen transfer for *Corydalis sheareri*. A horizontal orientation may be under selection of pollinators and co-adapt with other aspects of the inflorescence and floral traits.

## Introduction

Flower and inflorescence characteristics are thought to influence pollinator behavior [1]–[5]. Floral orientation, the angle between a flower’s main axis and the horizontal, is thought to affect pollinator attraction [6]–[10], foraging behavior [10]–[12], and pollen transfer [11], [13], [14]. However, most previous studies are on plant species that produce single flowers [8], [9], relatively little is understood about the effect of floral orientation on pollination in more complex inflorescences.

In vertical bee-pollinated inflorescences packaged in racemes or spikes, flowers typically have a horizontal orientation [15] This trait is explained by hypotheses regarding pollinator behaviors and pollination precision. Firstly, pollinator recognition: The bumble bees were reported to have a preference for horizontal flowers [16]. Sprengel (1793) proposed that bees are mainly attracted to horizontal-facing flowers from the front, and forcing this course of approach may facilitate the recognition of flower patterns [17], [18]. The inflorescences with horizontal flowers are thought to receive more visits due to enhanced recognition. Secondly, consistency of pollinator movement on inflorescences: bees have a tendency to move upwards in vertical inflorescences [4], [19]–[22], and a horizontal orientation is thought to ensure consistency of pollinator movements on inflorescences [12]. Finally, pollination precision: A horizontal orientation may function to control the access of landing sites and ensure the contact between floral reproductive organ and pollinator body [17]. Deviation from horizontal may result in decreased pollen transfer. These hypotheses predicted the significance of floral orientation on plant reproductive success from different aspects. However, the effects of horizontal orientation on pollinator recognition and pollination precision have been investigated for relatively few plant taxa pollinated by certain pollinator groups, e.g. syrphid fly [11], hummingbird [13]. In addition, relatively less is known about the effect of floral orientation on pollinator attraction of inflorescences [13]. Moreover, experimental investigations are needed to directly test the effect of floral orientation on the consistency of pollinator movement on inflorescences.

In this study, we test the hypotheses by using a species of *Corydalis*, a genus characterized by zygomorphic, bee-pollinated, horizontal-facing flowers arranged in racemes [23]–[27]. By artificially manipulating the orientation of individual flowers to prepare inflorescences with different floral orientations, we attempt to detect whether change of floral orientation affects (1) the capacity of an inflorescence to attract pollinators, (2) the number of successive probes and consistency of pollinator movement on inflorescences, (3) efficiency of pollen removal, and (4) seed production. Since pollinator attraction is associated with pollen transfer and mate diversity [5], the movement of pollinators on vertical inflorescences would affect geitonogamy and pollen export [1], [4], [28]. We predict that a deviation from the horizontal (natural) orientation will result in decreases in both male and female fitness, due to declines in pollinator attraction and consistency of pollinator movement.

## Materials and Methods

### Ethics Statement

This study was conducted in accordance with all People’s Republic of China laws. No specific permits were required for the described field studies. No specific permissions were required for access to the locations described in this study. The location is not privately owned and neither is it protected in any way. This study species is also not protected by any law.

### Study Species and Site

*Corydalis sheareri* S. Moore f. *sheareri* is one of the most common spring ephemerals in China. Each plant generally produces 2 to 10 inflorescences, with flowers arranged in racemes. The flowers are protandrous, with the lower (older) flowers in the female stage and upper (younger) flowers in the male stage. There is a considerable overlap in anthesis among the flowers of a single inflorescence, and the display size mostly ranges from 1 to 10. The flowering period of an inflorescence ranges from 9 to 23 days. Each flower is attached at the side of a branch by a pedicel, and the flower display forms a one-sided raceme. The pollen production per flower is 20273.72±3712.72 and ovule number is 22.55±2.14 (N >30, mean ± SD). The pollen-ovule ratio (ranges 750–1067) of this species matches that of facultative xenogamy [29]. The flowers of this genus show secondary pollen presentation, and the pollen is released on stigmatic area before flowers open [24], [27], [30]. However, bagged or hand self-pollinated flowers yield no seeds, and the self-pollen tubes often do not reach the ovules (unpublished data), indicating that pollinator-mediated cross-pollination is required for successful seed and fruit set. The flowers have a near horizontal orientation that is on average −6.80° ±10.63 (mean ± SD) to the horizontal. The corolla has four petals, and the upper petal extends to form a spur in which nectar collects. The stigma and anthers are concealed in the inner petals, when legitimate pollinators visit from the front of the flowers, they depress the inner petals and expose the reproductive surfaces [24].

We conducted the study in a natural population located in Sandouping, Hubei Province, China (30° 49′ 46.3″ N, 111° 04′ 03.3″ E, 150 m in altitude). The population consisted of more than 1000 flowering individuals, forming mono-specific patches surrounding forest fragments. Flowering period began in March and lasted to late April. Seeds matured in May. In this population, bee species, primarily leafcutter bees (*Megachile* spp.) and bumblebees (mostly *Bombus richardsi*) are the major pollinators. *Veronica persica* and *Petasites tricholobus* also flower at the same time, but they are usually visited by syrphid flies and honeybees, respectively (personal observation). Our field study was conducted from 10 March to 5 May 2013, encompassing the flowering peak at the study site.

### Flower Manipulation

**Figure 1:**
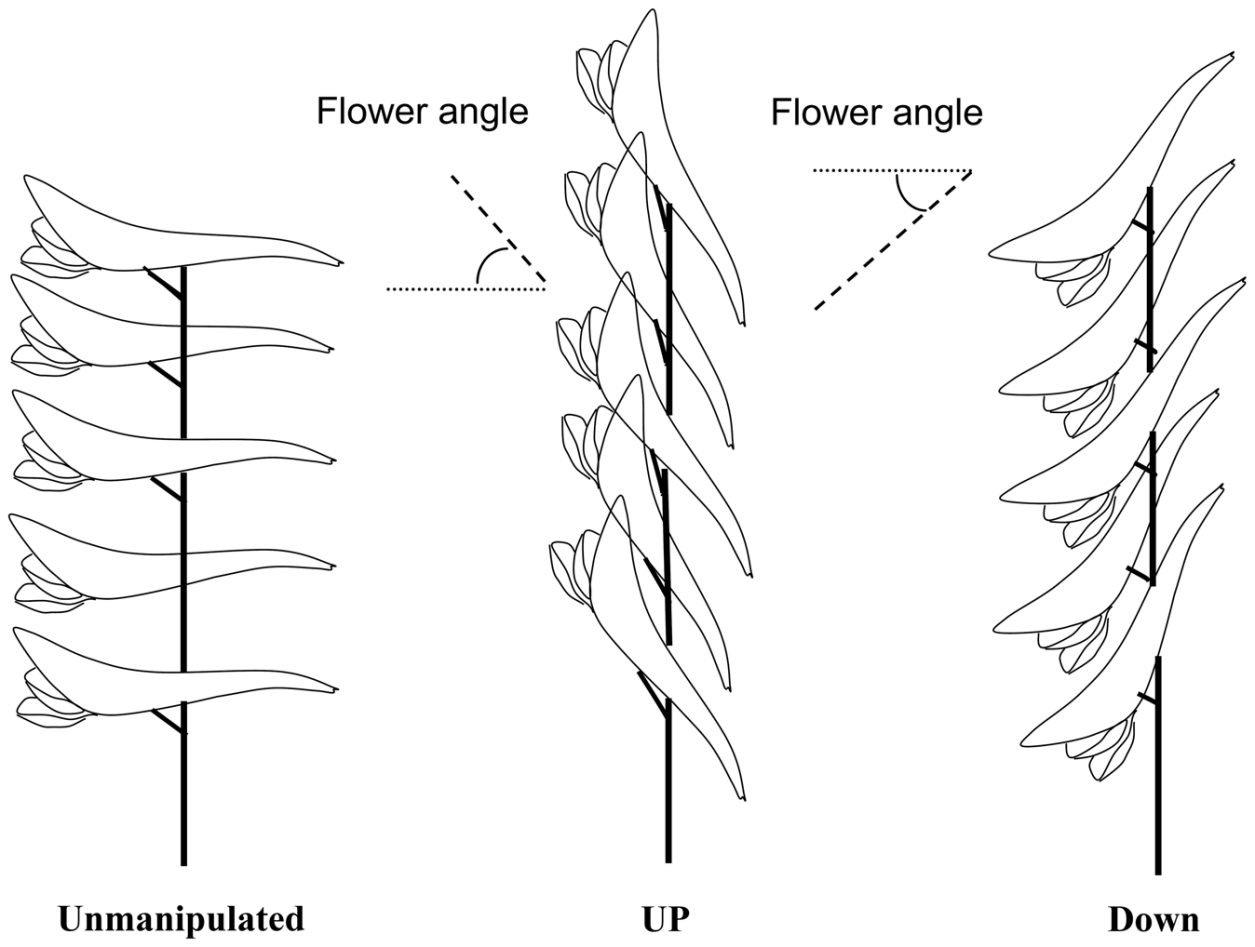
Side views of the three inflorescence types: horizontal (Unmanipulated), upward-facing (Up), and downward-facing (Down). The positive or negative angles between a flower’s main axis and the horizontal indicate the floral orientation. The flower angle was manipulated by leaning the pedicel of individual flowers and taping the terminal of the spur to the inflorescence stalk.

We presented three types of inflorescences with different floral orientations (Figure 1): (i) unmanipulated inflorescences with horizontal-facing flowers (Unmanipulated), (ii) inflorescences with flowers turned upward (Up), and (iii) inflorescences with flowers turned downward (Down). We conducted pollination observation by using individual flower patch. Each flower patch contained at least nine flowering individual plants and was 3 m in minimum separation between each other. Inside the patch, nine inflorescences with newly open flowers were selected from different plants and randomly assigned to the treatments (three of each type). The angle of individual flowers was altered by leaning the pedicel and taping the terminal of the spur to the inflorescence stalk. These manipulations were carefully conducted to ensure not to damage flower structure. We avoided shaking of flowers in order to not cause pollen loss. To minimize the influence of display size on pollinator behavior [4], [31], each studied inflorescence was trimmed to five flowers.

### Pollinator Observation

For each flower patch, we observed pollination on focal inflorescences for a period of 15 minutes. We conducted a total of 165 observation periods from 0800 h to 1600 h on sunny days when pollinators were active. Moreover, different flower patch was used for each observation period. We scored two types of pollinator behaviors on inflorescences: approaches and visits. An approach occurred when a pollinator found an inflorescence and approached it from the front side of the flowers. A visit occurred only when the pollinator probed at least one flower in an inflorescence. Each visit on an inflorescence was tracked and we recorded the number of successive probes within an inflorescence, as well as the direction (upward/downward) of movements between flowers of the same inflorescence. To detect changes of pollinator attraction with different floral orientation, we counted the number of inflorescences that were approached and visited per observation period. To detect changes of pollinator behavior within inflorescences, we used the number of successive probes within an inflorescence during a visit and the direction of vertical movements on an inflorescence.

### Pollen Removal and Seed Set

For each treatment (Unmanipulated, Up, Down), we measured pollen removal during a visit by using at least 30 enclosed inflorescences which were exposed to pollinators. The flowers were collected after they received a single visit from bees. We used individual flowers as replicates (N = 93 for leafcutter bees and N = 29 for bumble bees, respectively). Additionally, we randomly collected 20 flower buds to count pollen production per flower. All collected flowers were separately stored in vials containing 1 ml 70% ethanol.

Pollen removal was estimated as the number of pollen grains removed: *P*_0_–*P*_v_. *P*_0_ refers to pollen production per flower, and *P*_v_ is the number of pollen grains remaining on visited flowers. Since in *Corydalis*, pollen is secondarily presented on stigmas [30], the number of pollen grains left in anthers and on stigmas of the flower constitutes the number of pollen remaining in each visited flower. The anthers and stigmas were squashed to dislodge pollen grains, and the number of pollen grains in five 20-µL subsamples from each flower was counted under light microscopy (×4).

For each treatment (Unmanipulated, Up, Down), we measured seed production per inflorescence by using at least 20 enclosed inflorescences each from different plants. The inflorescences were trimmed to five flowers and exposed to pollinators. When the seeds matured, we counted seed production per inflorescence as the total number of seeds produced by each inflorescence. We harvested a total of 49 inflorescences to collect seed data, because some of the focal inflorescences were damaged in field.

### Data Analyses

Analyses of pollinator behavior were conducted for leafcutter bees and bumble bees separately, because pollinator taxa may differ in their foraging preference and direction of movement on inflorescences [16], [32]. We used the data from observation periods during which at least an approach was observed. To test whether a horizontal orientation facilitated pollinator recognition and attraction, we compared the number of inflorescences that were approached or visited per observation period among treatments (Unmanipulated, Up, Down), using observation periods as replicates. We applied generalized linear models (GLMs; with Poisson error and logarithmic link). The number of approached or visited inflorescences was treated as the response variable and the treatment as a fixed effect. To test the effect of floral orientation on occurrence of visits following approaches, we applied generalized linear mixed models (GLMMs) with binomial errors and logit-link function. The treatment was considered as a fixed effect and the response variable was the presence/absence (1/0) of a visit after an approach. Because the observation period was the source of replication and the approaches occurred in the same period were not independent of each other, we included observation period as a random term. We only used the data from inflorescences that received at least an approach. We used GLMMs (with Poisson error and logarithmic link) to examine the effect of floral orientation on the number of successive probes within an inflorescence per visit. The number of successive probes was considered as a response variable. The treatment was considered as a fixed effect and observation period as a random term. Only data from inflorescences received at least a visit was used. To analyze the consistency of pollinator movement on inflorescences, we compared the direction percentage of vertical movements (upward and downward) to the total vertical movements among treatments using Pearson chi-squared test.

We also compared the pollen removal per visit per flower and seed set per inflorescence among treatments using GLMs (with Poisson error and logarithmic link), using individual flowers and inflorescences as unit of replication, respectively. All statistical analyses were conducted in SPSS 19.0 at significant level of 0.05.

## Results

### Pollinator Behavior

Compared to the unmanipulated inflorescences, the number of inflorescences that were approached and visited by leafcutter bees decreased in both Up and Down treatments (Table 1). The approaches by bumblebees decreased only in the Up treatment, however, the number of inflorescence visited by bumble bees decreased for both Up and Down treatments (Table 1). Compared with Unmanipulated inflorescences, the occurrence of visits following approaches decreased in manipulated inflorescences for both pollinator types (GLMM, leafcutter bee, Up: *b* = −1.433±0.309, *z* = −4.638, *P*<0.001; Down: *b* = −0.698±0.275, *z* = −2.538, *P* = 0.011; bumble bee, Up: *b* = −2.455±0.669, *z* = −3.669, *P*<0.001; Down: *b* = −1.769±0.354, *z* = −4.997, *P*<0.001; Unmanipulated is used as the baseline and a negative value of *b* implies a negative effect of the treatment).

**Table 1:** Effects of changes in floral orientation on the number of inflorescences approached and visited per observation period (Mean ± SD) by leafcutter bees and bumble bees based on generalized linear model (GLM).

Pollinator type	Treatment	Mean ± SD	N^a^	*b*	*z*	*P*
Number of inflorescences approached per observation period						
Leafcutter bee	Up	0.65±1.04	93	−0.875±0.154^b^	−5.68	<0.001
	Down	1.04±1.77	93	−0.395±0.131	−3.02	0.003
	Unmanipulated	1.55±1.47	93	0^c^		
Bumblebee	Up	0.38±1.03	55	−0.847±0.261	−3.25	0.001
	Down	0.73±0.95	55	−0.203±0.213	−0.95	0.341
	Unmanipulated	0.89±0.94	55	0		
Number of inflorescences visited per observation period						
Leafcutter bee	Up	0.33±0.76	93	−1.311±0.202	−6.49	<0.001
	Down	0.66±1.29	93	−0.634±0.158	−4.01	<0.001
	Unmanipulated	1.24±1.11	93	0		
Bumble bee	Up	0.15±0.36	55	−1.682±0.385	−4.37	<0.001
	Down	0.40±0.53	55	−0.670±0.262	−2.56	0.011
	Unmanipulated	0.78±0.76	55	0		

a indicates numbers of observation periods (15 min).

b a negative value of *b* implies the treatment has a negative effect on variable.

c Unmanipulated is used as the baseline.

Leafcutter bees made 1.69±0.83 (mean ± SD), 1.59±0.50, 1.75±0.87 successive probes during a visit on Unmanipulated, Up and Down inflorescences. For bumble bees, the values were 1.74±0.98, 1.50±0.54 and1.29±0.46, respectively. The number of successive probes didn’t differ among treatments for leafcutter bees (GLMM, Up, *b* = −0.058±0.071, *z* = −0.817, *P* = 0.413; Down, *b* = 0.037±0.079, *z* = 0.468, *P* = 0.635). Compared with Unmanipulated inflorescences, the bumble bees made significantly less probes during a visit on Down inflorescences (Up, *b* = −0.151±0.141, *z* = −1.071, *P* = 0.286; Down, *b* = −0.305±0.118, *z* = −2.585, *P* = 0.010). Changes of floral orientation significantly affected the consistency of movement on inflorescences for leafcutter bees, as the proportion of upward movement was significantly greater in the horizontal than in the Up and Down inflorescences (Table 2). The direction of movement by bumble bees was not analyzed since their movements on Up and Down inflorescences were relatively infrequent.

**Table 2:** Effects of changes in floral orientation on the percentage of upward movements to total vertical movements on inflorescences by leafcutter bees and bumble bees.

Pollinator type	N^a^	Unmanipulated	Up	Down	χ^2^	*P*
Leafcutter bee	93	0.86^A^ (80)^b^	0.63^B^ (19)	0.57^B^ (46)	14.626	0.001
Bumblebee^c^	55	0.88 (32)	1.0	1.0		

Sites with different capital letters in the same row indicate significant differences among inflorescence types (P<0.05).

a indicates numbers of observation periods (15 min).

b number in bracket indicates the total number of vertical movements.

c data on bumble bees was not analyzed because the movements on Up and Down inflorescences were infrequent.

### Pollen Removal and Seed Set

**Figure 2:**
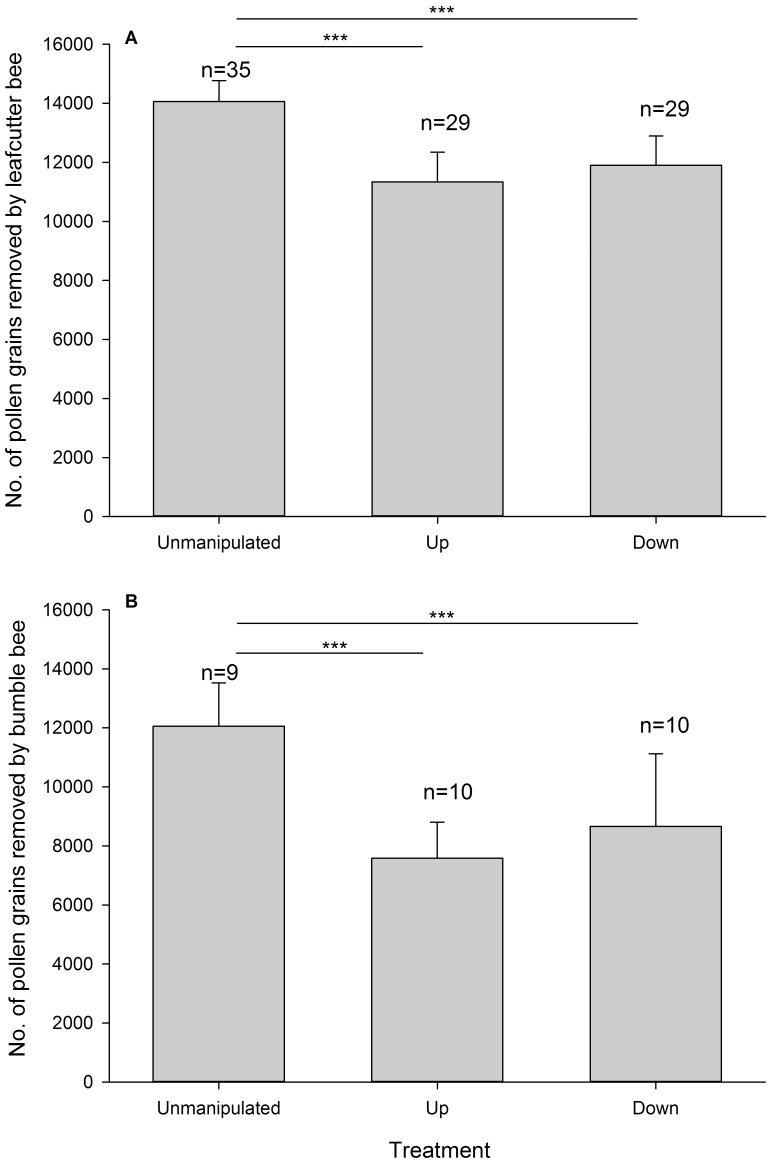
Comparisons in number of pollen grains removed after a single visit per flower by leafcutter bees (A) and bumble bees (B) among unmanipulated, UP, and DOWN inflorescences. Bars show standard errors. ***P<0.001.

The leafcutter bees removed 69.34±20.78% of pollen per flower per visit from Unmanipulated inflorescences, 55.91±26.90% from Up inflorescences and 58.73±26.28% from Down inflorescences. For bumble bees, the values for Unmanipulated, Up and Down inflorescences were 59.47±21.72%, 37.41±19.10% and 42.72±38.40%, respectively. Both pollinator types removed significantly less pollen grains per visit from Up and Down inflorescences, relative to Unmanipulated inflorescences (GLM, leafcutter bee, Up: *b* = −0.215±0.002, *z* = −107.500, *P*<0.001; Down: *b* = −0.166±0.002, *z* = −83.000, *P*<0.001; bumble bee, Up: *b* = −0.464±0.005, *z* = −92.800, *P*<0.001; Down: *b* = −0.331±0.005, *z* = −66.200, *P*<0.001; Figure 2).

**Figure 3:**
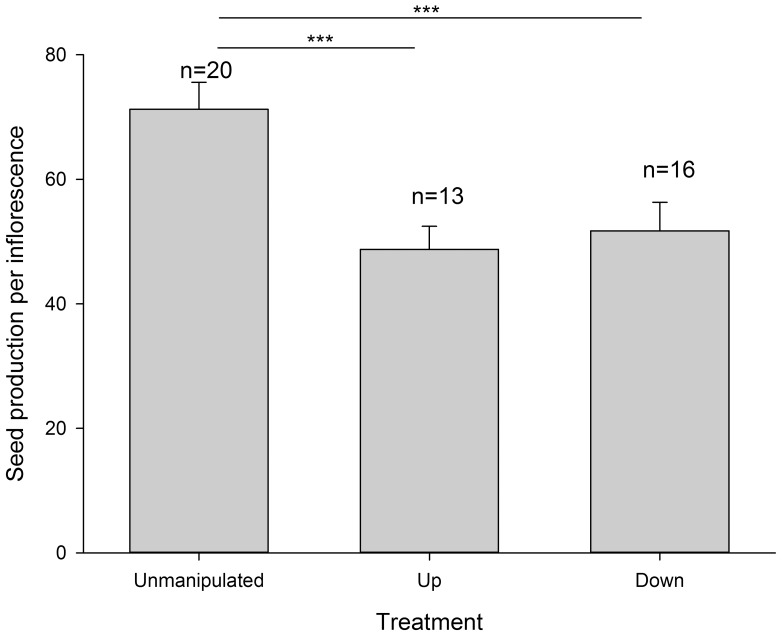
Comparisons in seed production per inflorescence among unmanipulated, UP, and DOWN inflorescences. Bars show standard errors. ***P<0.001.

Additionally, deviation from the horizontal orientation also significantly decreased seed production per inflorescence (GLM, Up: *b* = −0.379±0.048, *z* = −7.896, *P*<0.001; Down: *b* = −0.320±0.044, *z* = −7.273, *P*<0.001; Figure 3).

## Discussion

We tested three hypotheses concerning the role of horizontal orientation in affecting pollinator behavior and pollen transfer. These hypotheses predict higher pollinator attractiveness, higher pollination efficiency and greater consistency of pollinator movement for inflorescences with horizontal-facing flowers. Our results using bee-pollinated *C. sheareri* inflorescences support the three hypotheses.

For the manipulated inflorescences, the decreases in approaches indicate that bee recognition of the floral pattern reduced. The decreases in occurrence of visits following approaches indicate that changing floral orientation may impair the function of floral landing platforms. In addition, the declines in pollen removal and consistency of bee movement may be attributed to changes in postures when bees foraged on flowers of different orientation.

Legitimate pollinators typically approached the one-sided racemes and foraged on the horizontal-facing zygomorphic flowers from the front. Altering floral orientation could affect visibility of floral patterns and impair the function of floral landing platforms [17], [18], resulting in decreased pollinator recognition and diminished inflorescence attractiveness. The argument was supported by our results that changes in floral orientation led to significant decreases in the number of approaches and visits to inflorescences. The inflorescences with downward-facing flowers received as many approaches by bumble bees as the unmanipulated inflorescences, suggesting bumble bee recognition of foraging opportunities may not be affected by turning flowers downwards. This is consistent with the fact that bumble bees do specialize on some plant species with downward flowers [33], [34], and may not dislike downward-facing flowers [8]. However, the decreases in visits after approaches for inflorescences with downward flowers indicate that downward flowers may have a reduced attractiveness. Hummingbirds which specialize on plant species with pendant flowers were also reported to have a tendency to prefer less-pendent flowers to pendent ones [13]. This was explained that horizontal flowers may be more visible and accessible because their entrances are displayed in the surface of the inflorescence [13]. Additionally, downward orientation is also a character of wilting flowers in some plant species, including *C. sheareri*. Since wilted flowers are presumably less rewarding [35], [36], inflorescences composed of downward flowers are not as attractive as horizontal flowers.

Leafcutter bees typically move upwards on an inflorescence, as has been shown by Iwata et al. (2012). Racemes enhance greater consistency of pollinator movement compared to panicles and umbels [1]. Nevertheless, changes in floral orientation significantly increased the proportion of downward movements by leafcutter bees, supporting the hypothesis that a horizontal orientation ensures consistency of pollinator movement on inflorescences. Corbet et al.(1981) proposed that foraging posture may affect the direction of pollinator movement. Pollinators usually fly and forage in an upright position [15], [20], [21], and moving upwards rather than turning around is considered to incur less cost in time and energy [21]. This is corresponding with the fact that pollinators who forage in a head-down position tend to move downwards on inflorescences [21]. Additionally, the upward movement is also explained by that pollinators may have a better vision above than below when they position themselves upright [20], [37]. The effect of floral orientation on direction of pollinator movement may be attributed to the influences on both foraging posture and the view of pollinators. When pollinators forage on horizontal or downward-facing flowers, they generally maintain an upright position [12], [20], [38]. Therefore, the pollinators would have a better view of entrance to flowers above [20], and tend to move upwards rather than turning around. By contrast, pollinators forage on upward-facing flowers in a head-down position [12], they would have a better view of flowers below and probably make more downward movements. Inconsistent with these ideas, we found that the proportion of downward movements increased in both artificially Up and Down inflorescences. Interpretation is complicated by the fact that altering the floral orientation also altered the pollinator behavior when they switched between flowers within the inflorescences. The pollinators can only fly to next flower when flowers face horizontal, but changing floral orientation decreased the space between neighboring flowers and enabled pollinators to crawl between neighboring flowers. A higher directionality of upward movement is predicted in flights because insects may have a better control of their flight when they move against gravity [28]. By contrast, inflorescence characters that facilitate crawling between flowers would reduce the consistency of pollinator movement [12], and crawling between neighboring flowers would reduce flight from one flower to another [22]. Therefore, we propose that the effect of floral orientation on consistency of pollinator movement may be associated with flower arrangement.

Floral orientation is thought to affect the efficiency of pollen transfer due to changes in pollinator landing behavior or mechanical fit between plant reproductive organs and pollinators [10], [11], [39]. Foraging on downward flowers requires bees to remain in hanging position and foraging on upward flowers requires a head-down position. Though both leafcutter bees and bumblebees are relatively skilled to forage on *C. sheareri* flowers with different orientations, the changes in foraging posture may cause variations in foraging behavior and the fit between bees and floral reproductive organs.

Since the pollinator attraction is associated with pollen transfer and mate diversity, reductions on attraction may reduce both male and female functions [5]. The movement of pollinators on inflorescences may mediate pollen transfer, affecting geitonogamy and pollen export [1], [5], [28]. In our study, bumble bees made less successive probes on inflorescences with downward-facing flowers, relative to unmanipulated inflorescences. However, the directions along which pollinators move usually combine with the direction of dichogamy and order of development in inflorescences, affecting geitonogamous self-pollination and pollen-stigma interference [1], [28]. In protandrous racemes of *C. sheareri*, lower (older) flowers always display female function while upper (younger) flowers are in the male stage. The upwards direction of bee movements functions to facilitate outcrossing, because the pollinators start to visit lower stigma-presenting flowers and deposit cross-pollen from previously visited plants [28]. Meanwhile, pollen export can be enhanced as the pollinators left the inflorescences from upper pollen-presenting flowers [28]. By contrast, a higher percentage of downward movements would result in more pollen transferred from higher (male) flowers to lower (female) flowers. For self-incompatible *C. sheareri*, though self-fertilization is impossible, the self-pollen may occupy the stigmatic area and restrict the deposition of cross-pollen [40], as well as reduce the growth of cross-pollen tubes [41]. Moreover, the amount of pollen transferred to other plants would be also reduced [1], [5], [42]. In our study, the decreased seed set in manipulated inflorescences may be the result of reduced pollinator attraction and pollination efficiency, as well as increased interference by self-pollen.

Functional aspects of floral orientation in pollinator behavior and pollination precision have been investigated in individual flowers. In our study, we confirmed that a horizontal orientation has adaptive significance in ensuring consistency of pollinator movement on inflorescences. Since manipulation of pollinator behavior to promote outcrossing and pollen export is critical in the evolution of floral and inflorescence characters [43], the orientation of flowers within inflorescences may be under selection by pollinators. Though inflorescence architecture and floral orientation are traits showing quite a bit of phylogenetic conservatism, our results verify that floral orientation may act together with certain inflorescence architecture in controlling pollinator movement. Further research is necessary to examine the effect of floral orientation on pollen flow within inflorescences, in order to understand the influences of a horizontal orientation on geitonogamy and outcrossed siring success. In addition, the generality of the effects of floral orientation on pollinator behavior calls for investigations in plants pollinated by different pollinator types, with different floral design and inflorescence architecture.

## References

[1] Jordan CY, Harder LD (2006) Manipulation of bee behavior by inflorescence architecture and its consequences for plant mating. Am Natur 167: 496–509. 16670993 10.1086/501142

[2] Glaettli M, Barrett SCH (2008) Pollinator responses to variation in floral display and flower size in dioecious *Sagittaria latifolia* (Alismataceae). New Phytol 179: 1193–1201. 18627490 10.1111/j.1469-8137.2008.02532.x

[3] Ishii HS, Hirabayashi Y, Kudo G (2008) Combined effects of inflorescence architecture, display size, plant density and empty flowers on bumble bee behaviour: experimental study with artificial inflorescences. Oecologia 156: 341–350. 18283497 10.1007/s00442-008-0991-4

[4] Iwata T, Nagasaki O, Ishii HS, Ushimaru A (2012) Inflorescence architecture affects pollinator behaviour and mating success in *Spiranthes sinensis* (Orchidaceae). New Phytol 193: 196–203. 21919912 10.1111/j.1469-8137.2011.03892.x

[5] Harder LD, Barrett SCH (1996) Pollen dispersal and mating patterns in animal-pollinated plants. In: Lloyd DG, Barrett SCH, editors. Floral Biology. Chapman& Hall, New York: Springer. 140–190.

[6] Kevan PG (1975) Sun-tracking solar furnaces in high arctic flowers: significance for pollination and insects. Science 189: 723–726. 17792542 10.1126/science.189.4204.723

[7] Kudo G (1995) Ecological significance of flower heliotropism in the spring ephemeral *Adonis ramosa* (Ranunculaceae). Oikos 72: 14–20.

[8] Huang SQ, Takahashi Y, Dafni A (2002) Why does the flower stalk of *Pulsatilla cernua* (Ranunculaceae) bend during anthesis? Am J Bot 89: 1599–1603. 21665586 10.3732/ajb.89.10.1599

[9] Patiño S, Jeffree C, Grace J (2002) The ecological role of orientation in tropical convolvulaceous flowers. Oecologia 130: 373–379. 28547043 10.1007/s00442-001-0824-1

[10] Ushimaru A, Hyodo F (2005) Why do bilaterally symmetrical flowers orient vertically? flower orientation influences pollinator landing behavior. Evol Ecol Res 7: 151–160.

[11] Ushimaru A, Dohzono I, Takami Y, Hyodo F (2009) Flower orientation enhances pollen transfer in bilaterally symmetrical flowers. Oecologia 160: 667–674. 19333624 10.1007/s00442-009-1334-9

[12] Fenster CB, Armbruster WS, Dudash MR (2009) Specialization of flowers: is floral orientation an overlooked first step? New Phytol 183: 502–506. 19422542 10.1111/j.1469-8137.2009.02852.x

[13] Tadey M, Aizen MA (2001) Why do flowers of a hummingbird-pollinated mistletoe face down? Funct Ecol 15: 782–790.

[14] Ushimaru A, Kawase D, Imamura A (2006) Flowers adaptively face down-slope in 10 forest-floor herbs. Funct Ecol 20: 585–591.

[15] Sprengel CK (1793) Das entdeckte Geheimnis der Natur im Bau und in der Befruchtung der Blumen. Verlag Wilhelm Engelmann, Leipzig.

[16] Gong YB, Huang SQ (2011) Temporal stability of pollinator preference in an alpine plant community and its implications for the evolution of floral traits. Oecologia 166: 671–680. 21253770 10.1007/s00442-011-1910-7

[17] Neal PR, Dafni A, Giurfa M (1998) Floral symmetry and its role in plant-pollinator systems: terminology, distribution, and hypotheses. Annu Rev Ecol Syst 29: 345–373.

[18] Giurfa M, Dafni A, Neal PR (1999) Floral symmetry and its role in plant-pollinator systems. Int J Plant Sci 160: S41–S50. 10572021 10.1086/314214

[19] Pyke GH (1978) Optimal foraging in bumblebees and coevolution with their plants. Oecologia 36: 281–293. 28309915 10.1007/BF00348054

[20] Waddington KD, Heinrich B (1979) The foraging movements of bumblebees on vertical “inflorescences”: an experimental analysis. J Comp Physiol 134: 113–117.

[21] Corbet SA, Cuthill I, Fallows M, Harrison T, Hartley G (1981) Why do nectar-foraging bees and wasps work upwards on inflorescences? Oecologia 51: 79–83. 28310313 10.1007/BF00344656

[22] Valtueña FJ, Ortega-Olivencia A, Rodríguez-Riaño T, Pérez-Bote JL, Mayo C (2013) Behaviour of pollinator insects within inflorescences of *Scrophularia* species from Iberian Peninsula. Plant Biol 15: 328–334. 22823112 10.1111/j.1438-8677.2012.00644.x

[23] Ohara M, Higashi S (1994) Effects of inflorescence size on visits from pollinators and seed set of *Corydalis ambigua* (Papaveraceae). Oecologia 98: 25–30. 28312792 10.1007/BF00326086

[24] Maloof JE (2000) Reproductive biology of a North American subalpine plant: *Corydalis caseana* A. Gray ssp. *brandegei* (S. Watson) GB Ownbey. Plant Species Biol 15: 281–288.

[25] Kudo G, Maeda T, Narita K (2001) Variation in floral sex allocation and reproductive success within inflorescences of *Corydalis ambigua* (Fumariaceae)- pollination efficiency or resource limitation? J Ecol 89: 48–56.

[26] Kudo G, Kasagi T (2004) Floral sex allocation in *Corydalis ambigua* populations visited by different pollinators. Ecoscience 11: 218–227.

[27] Zhang YW, Yu Q, Zhao JM, Guo YH (2009) Differential effects of nectar robbing by the same bumble-bee species on three sympatric *Corydalis* species with varied mating systems. Ann Bot 104: 33–39. 19465751 10.1093/aob/mcp104 PMC2706726

[28] Lloyd DG, Webb C (1986) The avoidance of interference between the presentation of pollen and stigmas in angiosperms I. Dichogamy. New Zealand J Bot 24: 135–162.

[29] Cruden RW (1977) Pollen-ovule ratios: a conservative indicator of breeding systems in flowering plants. Evolution 31: 32–46. 28567723 10.1111/j.1558-5646.1977.tb00979.x

[30] Stolle J (2004) Biological flora of Central Europe: *Corydalis pumila* (Host) Rchb. Flora 199: 204–217.

[31] Makino TT, Ohashi K, Sakai S (2007) How do floral display size and the density of surrounding flowers influence the likelihood of bumble bee revisitation to a plant? Funct Ecol 21: 87–95.

[32] Wyatt R (1983) Plant-pollinator interactions and the evolution of breeding systems. In: Real. L.ed. Pollination biology. Orlando. Academic Press. 51–95.

[33] Kobayashi S, Inoue K, Kato M (1997) Evidence of pollen transfer efficiency as the natural selection factor favoring a large corolla of *Campanula punctata* pollinated by *Bombus diversus* . Oecologia 111: 535–542. 28308115 10.1007/s004420050268

[34] Mahoro S (2003) Effects of flower and seed predators and pollinators on fruit production in two sequentially flowering congeners. Plant Ecol 166: 37–48.

[35] Klinkhamer PG, de Jong TJ (1990) Effects of plant size, plant density and sex differential nectar reward on pollinator visitation in the protandrous *Echium vulgare* (Boraginaceae). Oikos: 399–405.

[36] Gori DF (1989) Floral color change in *Lupinus argenteus* (Fabaceae): why should plants advertise the location of unrewarding flowers to pollinators? Evolution: 870–881. 10.1111/j.1558-5646.1989.tb05184.x 28564205

[37] Haynes J, Mesler M (1984) Pollen foraging by bumblebees: foraging patterns and efficiency on *Lupinus polyphyllus* . Oecologia 61: 249–253. 28309419 10.1007/BF00396768

[38] Sapir N, Dudley R (2013) Implications of floral orientation for flight kinematics and metabolic expenditure of hover-feeding hummingbirds. Funct Ecol 27: 227–235.

[39] Castellanos MC, Wilson P, Thomson JD (2004) ‘Anti-bee’ and ‘pro-bird’ changes during the evolution of hummingbird pollination in *Penstemon* flowers. J Evol Biol 17: 876–885. 15271088 10.1111/j.1420-9101.2004.00729.x

[40] Faegri K, Van der Pijl L (1979) The principles of pollination ecology. Pergamon Press, Oxford: 288.

[41] Howlett B, Knox R, Paxton J, Heslop-Harrison J (1975) Pollen-wall proteins: physicochemical characterization and role in self-incompatibility in Cosmos bipinnatus. Proc R Soc B 188: 167–182.

[42] Harder LD, Jordan CY, Gross WE, Routley MB (2004) Beyond floricentrism: the pollination function of inflorescences. Plant Species Biol 19: 137–148.

[43] Harder LD, Williams NM, Jordan CY, Nelson WA (2001) The effects of floral design and display on pollinator economics and pollen dispersal. Cognitive ecology of pollination. Cambridge University Press, Cambridge. 297–318.

